# Editorial: 15 years of frontiers in computational neuroscience - computational perception and cognition

**DOI:** 10.3389/fncom.2024.1531155

**Published:** 2025-01-07

**Authors:** Nicolangelo Iannella

**Affiliations:** Department of Biosciences, Faculty of Mathematics and Natural Sciences, University of Oslo, Oslo, Norway

**Keywords:** perception, cognition, spiking neurons, neural fields, memory capacity, manifold untangling, classification accuracy, memory model

The underlying mechanisms and corresponding neural circuits involved in how cognition and perception emerge in the brain have been actively studied for over 50 years. Fully understanding how these traits emerge is complex and challenging, leading to the development of novel tools, both experimental and computational. Over the last three decades, computational models have played important roles in investigating the complex action of neural circuits involved in perception and cognitive function, while developing a cellular and network-level description of how external information is transformed and perceived in the brain, in an orderly fashion. Despite the complex nature of such information, the brain can easily decode information through the coordinated activity of neural populations and corresponding processes. Here, computational models have proved useful for understanding such processes via building network models of neural dynamics, inspired by the mammalian cortex's hierarchical (deep) organization, which can mimic the activity of neural populations involved in some perceptual or cognitive task.

Fifteen years have passed since the inception of Frontiers in Computational Neuroscience, and this Research Topic is a celebration of the 15^th^ year anniversary of the journal as well as an opportunity for the community to highlight new results focusing on computational perception and cognition. This Research Topic, as a part of a series aimed at showcasing recent advances in the field of computational perception and cognition, provides an outlet to discuss current challenges and exciting new developments in computational perception and cognition. The presented articles on this Research Topic provide a snapshot of recent research outcomes that will hopefully inspire others to investigate the underlying neural correlates of cognition and perception.

Investigations presented in this Research Topic focus on several important aspects of perception and cognition, such as information coding and memory capacity. Wei and Li proposed that using directed graphs as abstractions of biological neural networks along with node-adaptive learning can encode, store, and retrieve information and further illustrated consistent memory performance that outperformed the Hopfield network in both memory retrieval accuracy and storage capacity.

While using a neural field model, Nesse et al. showed how embedded information in a visual stimulus can be represented in the dynamical signatures of neural activity which can be modulated by working memory signals. They illustrate how these signals enhance phase code discrimination by approximately two orders of magnitude, while simultaneously reducing rate code discrimination, suggesting a mechanism by which oscillatory signals can enhance sensory information. In line with this, Mysin presented a network model of the CA1 hippocampus with seven types of inhibitory neurons and illustrated novel phase relations with the theta rhythm, illustrating that the firing dynamics of interneurons are dominated by excitation.

For processing visual information, neurons in higher visual areas can dynamically change their responses, which can account for time-varying interactions between external visual stimuli and internal signals. This forms the basis of a behaviorally relevant representation space of the visual world. This space, however, has associated challenges for precisely quantifying individual contributions to the representation and for the readout of encoded sensory information during some behavioral tasks. In this Research Topic, Weng et al. reviews the generalized linear model (GLM) framework for providing a quantitative description of neural processing as a function of various input stimuli and links to particular behaviors for single trials and individual neurons, while acknowledging that most applications of GLMs assume that neural systems are time-invariant and thus lack the ability to mimic non-stationary characteristics of neuronal responses in higher visual cortical areas. Weng et al. focuses on time-varying extensions to GLMs and discusses their application for deciphering neural representations in higher visual cortical areas, decoding transient activity and linking this to behavioral responses by manipulating model components. This strategy enables the extraction of important insights for discovering fundamental computational principles that govern neuronal processing in higher visual areas and other brain regions while subject to various behaviors.

Furthermore, Xie and Sadeh focused on changes in neural activity in response to complex external stimuli caused by internal behavioral states and questioned whether and to what extent it was possible to predict the presented visual stimuli from activity across behavioral states and how this varies in different brain regions of the mouse brain. Using publicly available datasets from the Allen Brain Institute, they studied the performance of extracting visual information in awake mice by using decoders to examine whether neural activity recorded in different brain regions and under diverse behavioral states could determine natural movie frames based on local neural activity. Xie and Sadeh illustrated a spectrum of visual information present in various regions of the mouse brain, responding to binary and multiclass classification tasks. They illustrated the presence of a hierarchy of classification accuracies, with the visual cortical areas being the highest, followed by thalamic and midbrain regions and hippocampal areas being the lowest, where behavioral variability caused by switching between different stimulus sessions caused a decrease in decoding accuracy in the decoder's the classification performance.

Given the dynamic nature of how perception emerges in the brain, Purohit et al. have chosen to focus on visual-spatial perception as a process for extracting the spatial relationship between objects and how activity in the sympathetic or parasympathetic system can affect the internal representation of the external visual-spatial environment. Purohit et al. presented a quantitative model to investigate how hyperactivation- or hypoactivation-inducing agents can modulate visual-perceptual space. They found a nonlinear relationship between agent concentration and perceived alterations to visual space, based on the Hill equation, and this could be potentially used to screen for misjudgment in decision-making by highly stressed individuals.

Given that perception involves many functions including object recognition, Li and Wang proposed that the ventral stream, often associated with recognizing objects and faces, involves a novel mechanism called local subspace untangling based on a mathematical abstraction, based on the association of a manifold to an object category, to describe how the visual cortex carries out object recognition by distinguishing between different object categories through the disentanglement of their associated manifolds, noting that the problem of manifold untangling is closely related to the Schölkopf (2000) kernel trick, used in a support vector machine (SVM), which permits learning a non-linear function or decision boundary using linear learning algorithms, considered to be a technique that exploits the “blessing of dimensionality.” Li and Wang develops a general solution to manifold untangling for topological space without the need for a distance metric by employing the strategies of global manifold embedding in a higher dimensional space or local manifold flattening to promote selectivity or tolerance, respectively. Furthermore, Li and Wang discussed connecting such techniques to current studies on untangling image, audio, and language data and the application to internal and motor control representation.

A better understanding of cognition and perception permits novel applications to be designed. The example presented in this Research Topic by Roeder et al. focuses on using human memory encoding and recall of stimuli and categories to develop a prosthetic for the hippocampus. Roeder et al. built a new model that allows the hippocampus to encode specific items using spatiotemporal firing of neural populations that have encoded specific content into short-term memory. The resulting memory decoding model (MDM) of neural firing in CA3 and CA1 regions of the hippocampus derives a corresponding stimulation pattern that is then applied to these regions during the encoding phase of a delayed match-to-sample (DMS) human short-term memory task. They found that using MCM, memory encoding of specific content can be facilitated for patients with impaired memory function and thus potentially offers a stimulation-based method for developing an implantable neural prosthetic for improving human memory.

Finally, continued research in perception and cognition is important for addressing current and future challenges that can be applied to develop future applications in various areas such as medicine and autonomously engineered systems. This Research Topic gathers important recent developments in computational perception and cognition with a promising perspective on developing the next generation of tools and applications.

In conclusion, I hope that the work collected in this Research Topic will serve as a basis for future studies exploring the neural correlates of perception and cognition along with their potential applications.
